# Roles of parasympathetic outflow and sympathetic outflow in the cardiovascular response to brief umbilical cord occlusion in fetal sheep

**DOI:** 10.1371/journal.pone.0254155

**Published:** 2021-07-06

**Authors:** Morgan Recher, Arthur Lauriot Dit Prevost, Dyuti Sharma, Julien De Jonckheere, Charles Garabedian, Laurent Storme

**Affiliations:** 1 Univ. Lille, ULR 2694 – METRICS: Evaluation des technologies de santé et des pratiques médicales, Lille, France; 2 Department of Pediatric Intensive Care Unit, CHU Lille, Jeanne de Flandre Hospital, Lille, France; 3 Department of Pediatric Surgery, CHU Lille, Jeanne de Flandre Hospital, Lille, France; 4 CHU Lille, Centre d’Innovation Technologique, Lille, France; 5 Department of Obstetrics, CHU Lille, Jeanne de Flandre Hospital, Lille, France; 6 Department of Neonatology, CHU Lille, Jeanne de Flandre Hospital, Lille, France; University of Washington, UNITED STATES

## Abstract

Fetal heart rate (FHR) deceleration is the most common change seen during labor. The role of the autonomic nervous system in regulating the fetal cardiovascular response during multiple uterine contractions has been well-established. However, the mechanism underlying the hemodynamic response remains unclear and the specific reflex that mediates the cardiovascular modifications is still controversial. This study aimed to determine the role of the sympathetic and parasympathetic systems on fetal hemodynamics in complete cord occlusion. Chronically instrumented fetal sheep were randomized to receive an intravenous injection of atropine 2.5 mg (n = 8), propranolol 5 mg (n = 7), atropine and propranolol (n = 7), or a control protocol (n = 9), followed by three episodes of 1-minute umbilical cord occlusion repeated every 5 minutes. Cord compression induces a rapid decrease in the FHR and a rapid increase in MAP. The decrease in FHR is caused by an increase in parasympathetic activity, (atropine and atropine-propranolol abolish the FHR response to the occlusion). The change in FHR during occlusion was not modified by propranolol injection, showing no effect of sympathetic tone. The increase in MAP during occlusion was similar in the four protocols. After releasing occlusion, the FHR was still lower than that at baseline due to a sustained parasympathetic tone. Suppression of the parasympathetic output to the cardiovascular system unmasks an increase in the FHR above baseline values. The lower FHR with the propranolol protocol further supports an increase in myocardial β-adrenoceptor stimulation after cord release. The increase in MAP after cord release was similar in the four protocols, except after the early stage of interocclusion period in atropine protocol. Four minutes after cord release, the FHR returned to baseline irrespective of the drugs that were infused, thereby showing recovery of ANS control. Blood gases (pH, PaCO_2_, PaO_2_) and plasma lactate concentrations was similar between the four protocols at the end of three applications of UCO. Complete cord compression-induced deceleration is likely due to acute activation of parasympathetic output. β-adrenoceptor activity is involved in the increase in FHR after cord release. Understanding the reflexes involved in FHR deceleration may help us understand the mechanisms underlying fetal autonomic adaptation during cord occlusion.

## Introduction

During labor, fetal heart rate (FHR) recordings are the only non-invasive tool that can continuously monitor intrapartum fetal well-being. Knowledge and understanding of fetal physiology are required to interpret FHR recordings, [1,2]. Decelerations associated with uterine contractions are the most common and distinctive component of intrapartum FHR [3,4]. The role of the autonomic nervous system (ANS) in regulating FHR and mean arterial pressure (MAP) during uterine contractions to maintain optimal organ perfusion is well-established [3,5,6]. Heart rate (HR) is controlled by parasympathetic activity through M2 muscarinic receptors on nodal cells and by sympathetic activity mediated by ß1-adrenergic receptors on cardiac pacemaker cells [5]. However, the parasympathetic output and sympathetic output at different time points during and after FHR deceleration remain incompletely understood [3,7,8]. A better understanding of the sequence of ANS activity during FHR may help decipher which reflexes trigger FHR decelerations [9].

Experimental models have shown that brief umbilical cord occlusion (UCO) induces acute FHR deceleration and increased MAP, followed by an increase in FHR when the occlusion has been released [5,10]. This can occur during labor in cases of cord prolapse. Therefore, we hypothesized that changes in FHR during and after brief UCO depend on activation of both branches of the ANS.

Using cholinergic blockade and β1-adrenergic blockade drugs, we aimed to determine the roles of the parasympathetic and sympathetic nervous systems in changes in FHR and MAP during UCO in chronically instrumented near-term fetal sheep.

## Materials and methods

### Ethics

The anesthesia, surgery, and experimentation protocols were in agreement with the recommendations of the Ministry of Higher Education and Research of France. The study was approved by the Animal Experimentation Ethics Committee (CEEA #2016121312148878). All efforts were made to minimize suffering.

### Surgery and instrumentation

This study was conducted between November 2018 and June 2019. Fifteen pregnant “Ile de France” sheep (National Institute for Agronomic Research, INRA, France) underwent hysterotomy at 125±3 days of gestation (term = 140–145 days) under general anesthesia. Anesthetic and surgical procedures have been described previously [11,12]. Before surgery, sheep were placed in the supine position, anesthetized with inhaled isoflurane and oxygen, and intramuscular xylazine 0.1 mL/10 kg (Sedaxylan^®^; CEVA Santé Animale, Liboune, France, half-life 30 minutes), intubated, and maintained with isoflurane 2% (IsoFlo^®^; Zoetis, Torce, France) and oxygen. After an abdominal incision, hysterotomy was performed as previously described. The fetus underwent *in utero* surgery. In case of twins, only one twin was instrumented. The fetus received an intramuscular injection of buprenorphine 0.3 mL (Buprénodale^®^; Dechra Veterinary Products, Montigny-le-Bretonneux, France, half-life 6 hours) and a subcutaneous injection of lidocaine hydrochloride 1 mL (Xylocaïne^®^; Astra Zeneca, Courbevoie, France, half-life 2 hours) after hysterotomy. The fetal right and left upper limbs were successively brought out of the uterus for catheter insertion after a bilateral axillary incision (Vygon, Ecouen, France). Two aortic catheters were inserted via the right and left axillary arteries, with one positioned in the ascending aorta, for blood sampling and arterial pressure measurements, respectively. To confirm accurate placement of the catheter, we moved the catheter back 1 cm until extrasystoles ceased. The arterial pressure was then checked to ensure that the catheter correspond with the aortic pressure rather than the ventricular pressure. Furthermore, arterial pressure is measured during surgery to ensure that the tip of the catheter is inside the aorta and not in the left ventricle. The position of the catheter is also checked during autopsy. A venous catheter was inserted into the superior vena cava for drug infusion via the right axillary vein. Four subcutaneous electrodes (two electrodes per axillary incision) were sutured to the intercostal muscles for electrocardiographic monitoring (Mywire 10; MACQUET, Rastatt, Germany). An inflatable occluder was placed around the umbilical cord (OC16; In Vivo Metric, Healdsburg, California, USA). A 5-Fr catheter was placed inside the amniotic cavity to monitor intra-amniotic pressure (IAP). The catheters, electrodes, and occluder were exteriorized to the ewe’s flank and secured in a protective bag. Fetuses were then returned into the uterine cavity. During closure of the hysterotomy, an amniotic infusion of 250 mL of isotonic saline serum containing 500 mg of amoxicillin + clavulanic acid (Augmentin Intravenous, 1 g/200 mg, GlaxoSmithKline, Barnard Castle, United Kingdom) was administered through the 5-Fr catheter left in the intra-amniotic cavity to obtain a reference pressure. Postoperative analgesia of ewes was administered by an intravenous injection of 20 mg of nalbuphine, repeated 6 h after the first injection, and then daily until the third day after surgery. Similarly, fetal antibiotic prophylaxis with 500 mg of amoxicillin + clavulanic acid, and fetal analgesia with 10 mg of nalbuphine were administered daily through the intra-amniotic catheter until the third day after surgery. Catheters were maintained by daily injections of 2 mL of heparinized (10 IU/mL) normal saline. Fetuses were continually connected to the ewes during experiments and during the recovery periods between each experiment. Fetuses and ewes were monitored to check hemodynamic parameters until the end of the experimental period. Ewes were monitored and evaluated by a veterinarian. If the ewes showed any sign of pain, distress, or infection (modification of clinical signs, behavior changes), analgesics and antibiotics were rapidly prescribed and administered by a veterinarian. If the analgesic or antibiotic therapy was ineffective, the ewes were killed. If the fetus died or showed signs of suffering during the stability period, ewes were killed. At the end of the experimental procedures, animals were killed using T61^®^ (Tanax1, Intervet, Beaucouzé, France) IV infusion (3 mL for 10 kg body weight for the ewe and 0.3 mL/kg for the fetus).

No ewes died before or during experiments. Fifteen fetuses were operated upon, two died just after surgery, and one fetus was killed because the inflatable occluder was non-functional.

### Experimental procedure

Experiments began four days after surgery. One protocol was tested per day to allow for fetal recovery. Four protocols were designed to assess the mechanisms involved in FHR deceleration: UCO without any injected drugs (control protocol, n = 9); UCO after parasympathetic blockade by an injection of atropine (n = 8); UCO after β-adrenergic blockade by an injection of propranolol (n = 7); and UCO after both parasympathetic and β-adrenergic blockade with atropine and propranolol (atropine-propranolol; n = 7). The control protocol was performed first, and the order of the other protocols was randomized (Package “Blockrand” for R software). Drug concentrations were established according to previous work as 2.5 mg atropine sulfate (2.5 mg/2.5 mL; Atropine; Aguettant, Lyon, France) and 5 mg propranolol hydrochloride (5 mg/5 mL propranolol; Karnodyl; Primius Lab Ltd, London, United Kingdom) [5,11]. Both were administered as a bolus 5 minutes before the first UCO. Each experiment consisted of three applications of UCO for 1 minute with a recovery period of 4 minutes between each UCO, as described previously [5,11,13]. Prior to injection, an initial 30-minute period, called the stability period, was recorded. Total UCO was performed by rapid and complete inflation of the occluder using saline. If the ewe or fetus died or if problems occurred during the experiments, recordings have been excluded and then additional ewes were included to guarantee a minimum of seven experiments per protocol.

### Fetal hemodynamic parameters and blood samples

The FHR, MAP, and IAP (IntelliVue MX700; Philips, Eindhoven, Netherlands) were continuously monitored and recorded using a data acquisition board (Physiotrace©; Estaris Monitoring, Lille, France) during the study period [14]. MAP refers to the amniotic cavity pressure (MAP = observed MAP—observed IAP). We defined the percentage changes in FHR and MAP from baseline were calculated as (time point value—baseline value) × 100 / baseline value and were described as “change in FHR” and “change in MAP”, respectively. The baseline is the value obtained just before the occlusion. FHR, MAP, change in FHR, and change in MAP during UCO were recorded with a resolution of 1 second. Moreover, we defined six time points, averaged over 10 seconds, to compare change in FHR and change in MAP to baseline: just before UCO (baseline); then at 10, 30, 60, 120, and 300 seconds after starting UCO. To consider the intra-individual variability in the hemodynamic response to UCO, we averaged the values of the 3 UCOs to account for intraindividual variability in the hemodynamic response to UCO. In the case of outliers due to technical problems, these values were not taken into account during the analysis. Blood gases (pH, PaCO_2_, PaO_2_) and plasma lactate concentrations were measured from blood samples taken from the ascending aorta at baseline before drug infusion and then adjusted to the ewe’s physiological temperature of 39°C (i-Stat Handheld©; Abbott Laboratories, Washington, DC, USA). In addition, blood gas analyses and measurement of plasma lactate concentration were performed at the end of the three UCOs.

### Statistical analysis

We have compared FHR, MAP, change in FHR and change in MAP at each second time point by two-way ANOVA with time treated as a repeated measure and treatment as the independent factor was performed. The time epochs were analysed every minute. If a significant group effect was found, the Fisher’s protected least significant difference (LSD) post-hoc test was additionally performed. Individual time points were additionally tested by one-way ANOVA with group as the independent factor. To compare the averaged values at 10, 30, 60, 120, and 300 seconds after starting UCO, a non-parametric Friedman test was performed for repeated measures. When statistical significance was found, post hoc comparisons were performed using a Wilcoxon signed-rank non-parametric test with Bonferroni correction to compare time-point to baseline. A Kruskal-Wallis test were used for testing nonparametric data, post-hoc analysis was performed with a Dunn’s test with Bonferroni correction. A value of p < 0.05 was considered significantly different. In each experiment, *n* represents the number of studied protocols. Data are expressed as mean ± standard error. Statistical analyses were performed using SPSS (v26, SPSS, Chicago, IL) and R software version 3.4.1 (available online).

## Results

### Hemodynamics parameters

#### Before cord occlusion

Before drug infusion, baseline fetal arterial blood gases (pH, PaCO_2_, PaO_2_), plasma lactate concentrations, and fetal hemodynamic parameters (FHR, MAP) did not differ between protocols (Table 1). Fetal weights were similar with each protocol. After drug injections and before UCO, FHR was higher with the atropine protocol than with the control protocol, and lower with the propranolol protocol than with the control protocol (p<0.001).

**Table 1:** Fetal arterial blood gases and fetal hemodynamic variables before occlusion.

	Control	Atropine	Propranolol	Atropine-propranolol	p-value
**No. of protocols**	9	8	7	7	
**Blood gases**					
pH	7.39 (7.37–7.40)	7.39 (7.39–7.40)	7.38 (7.37–7.40)	7.39 (7.37–7.41)	0.83
PaCO_2_ (mmHg)	46 (43–47)	47 (42–48)	47 (42–48)	45 (41–46)	0.99
PaO_2_ (mmHg)	18 (17–20)	18 (17–21)	18 (15–20)	21 (18–21)	0.93
Lactates (mmol/L)	1.6 (1.6–1.9)	1.6 (1.2–2.1)	1.7 (1.3–2.9)	1.5 (1.1–1.8)	0.40
**Fetus variables**					
Weight (kg)	3.5 (3.1–4.0)	3.2 (3.1–3.9)	3.4 (3.1–3.9)	3.2 (3.1–3.8)	0.60
Heart rate before injection	164 (159–169)	171.00 (168–178)	153 (150–164)	165 (158–174)	0.11
Heart rate before occlusion	162 (153–173)	209 (192–236)*	130 (119–148)*	172 (161–179)	**<0.001**

Data are expressed as median interquartile range (IQR; first–third interquartile). p-value: Kruskal-Wallis test.

*p<0.05: Dunn’s test with Bonferroni correction (compared to control).

#### During cord occlusion (Figs 1 to 5)

**Fig 1:**
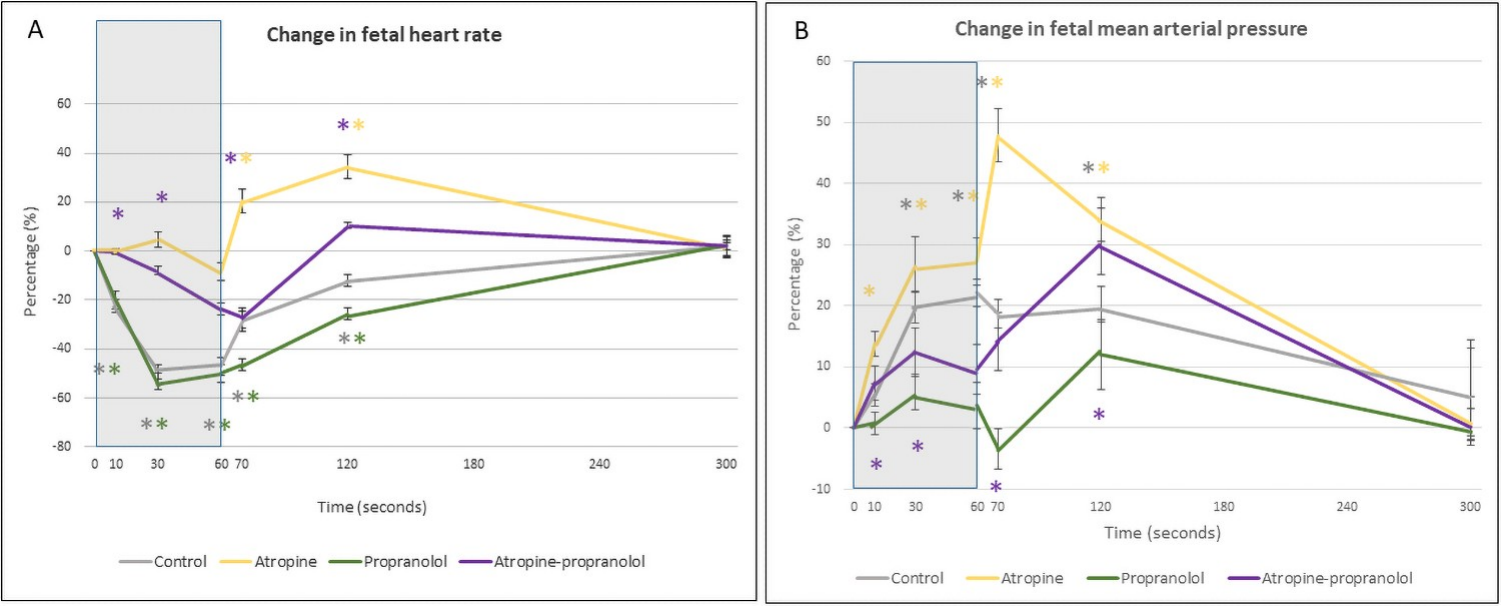
Change in fetal hemodynamics during and after cord occlusion. Change in fetal heart rate (A) and fetal mean arterial pressure (B) in treated fetuses exposed to three applications of 1-minute umbilical cord occlusion (grey zone) every 5 minutes. Data are expressed as mean ± standard error. *p < 0.05 vs. baseline.

**Fig 2:**
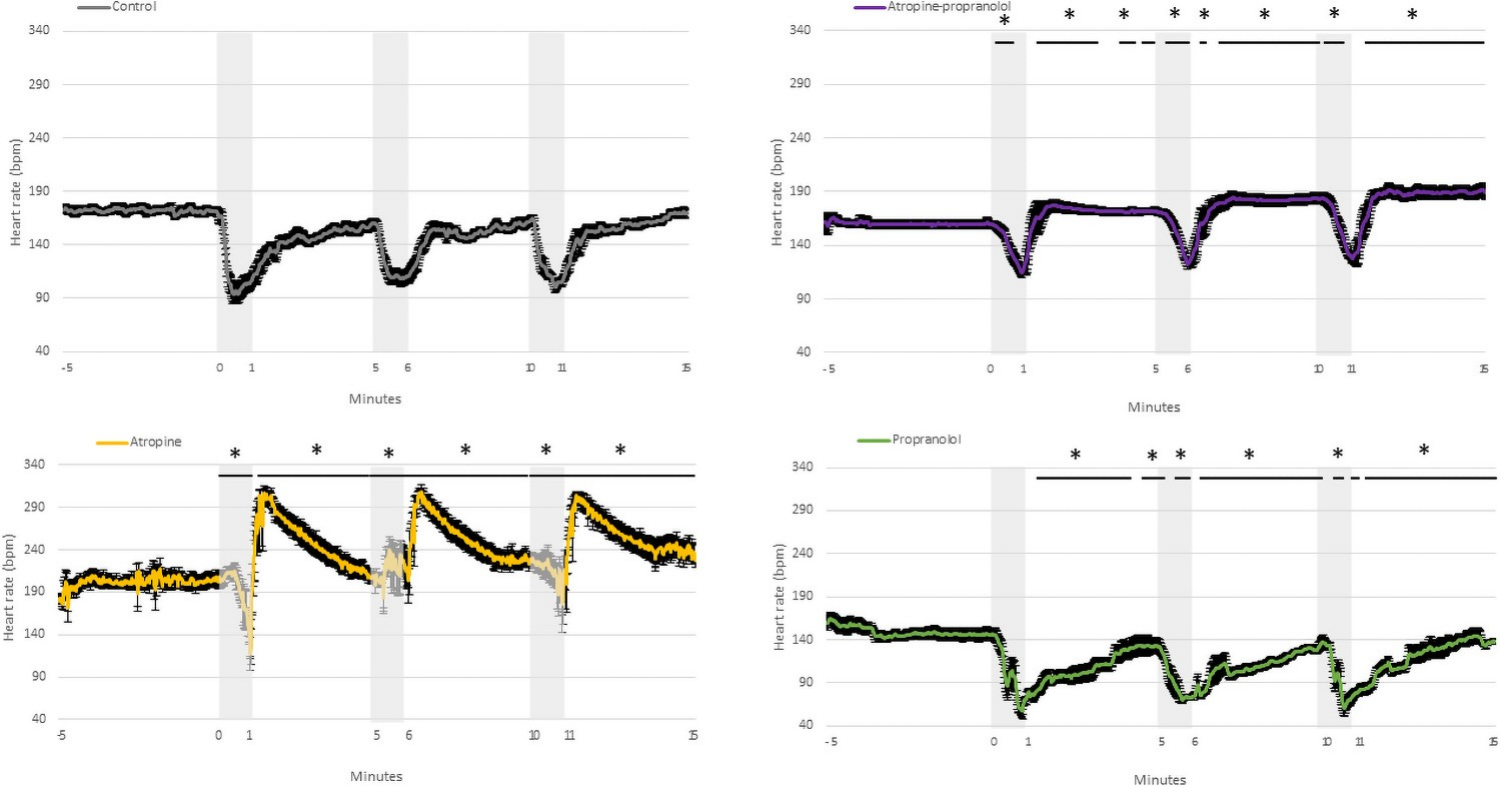
Fetal heart rate. Fetal heart rate in control, atropine, propranolol and atropine-propranolol protocols in fetuses exposed to three applications of 1-minute umbilical cord occlusion (grey zone) every 5 minutes. Data are expressed as mean ± standard error. *p < 0.05 vs. control. Resolution of each datapoint: 1 second.

**Fig 3:**
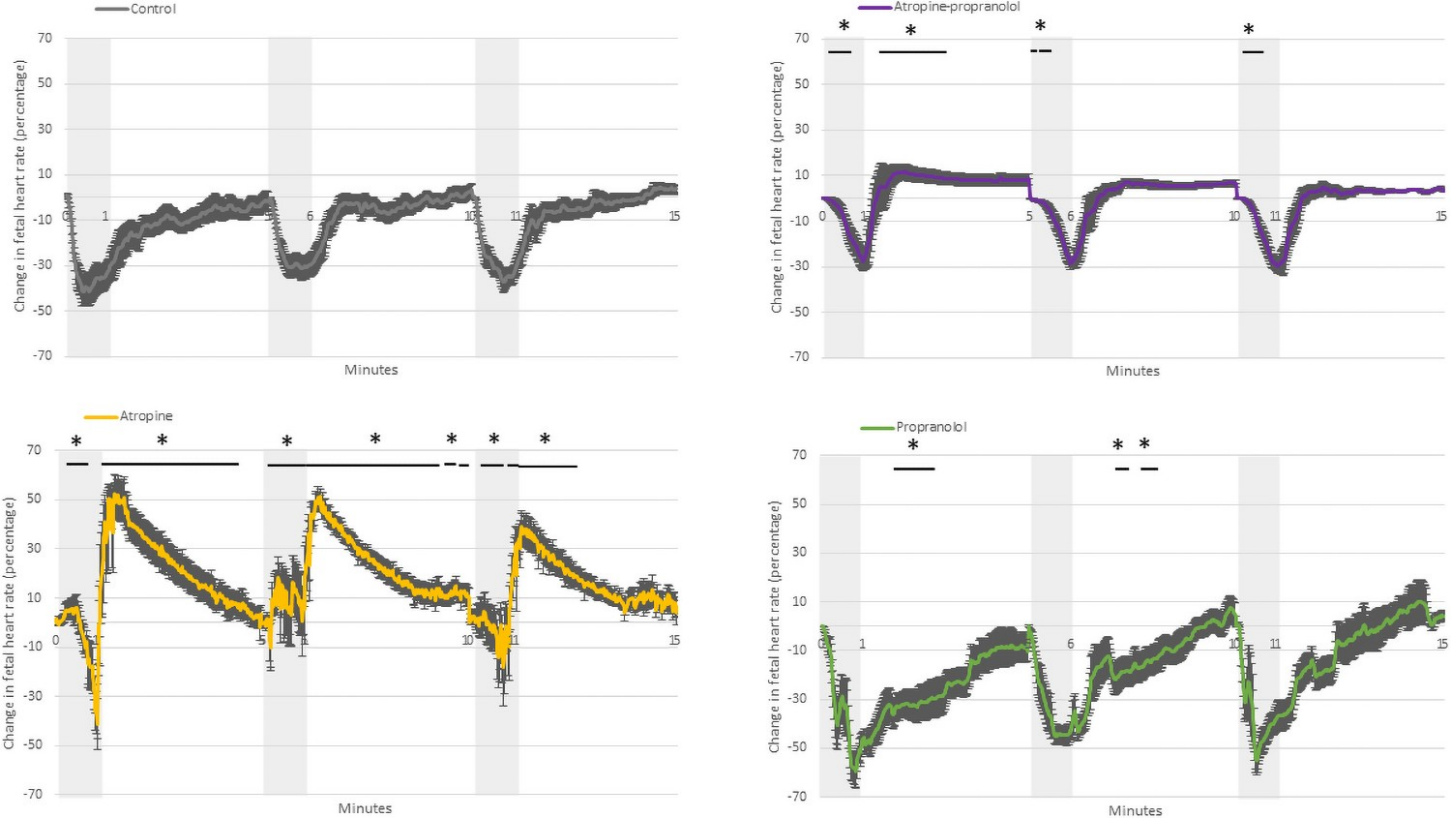
Fetal mean arterial pressure. Fetal mean arterial pressure in control, atropine, propranolol and atropine-propranolol protocols in fetuses exposed to three applications of 1-minute umbilical cord occlusion (grey zone) every 5 minutes. Data are expressed as mean ± standard error. *p < 0.05 vs. control. Resolution of each datapoint: 1 second.

**Fig 4:**
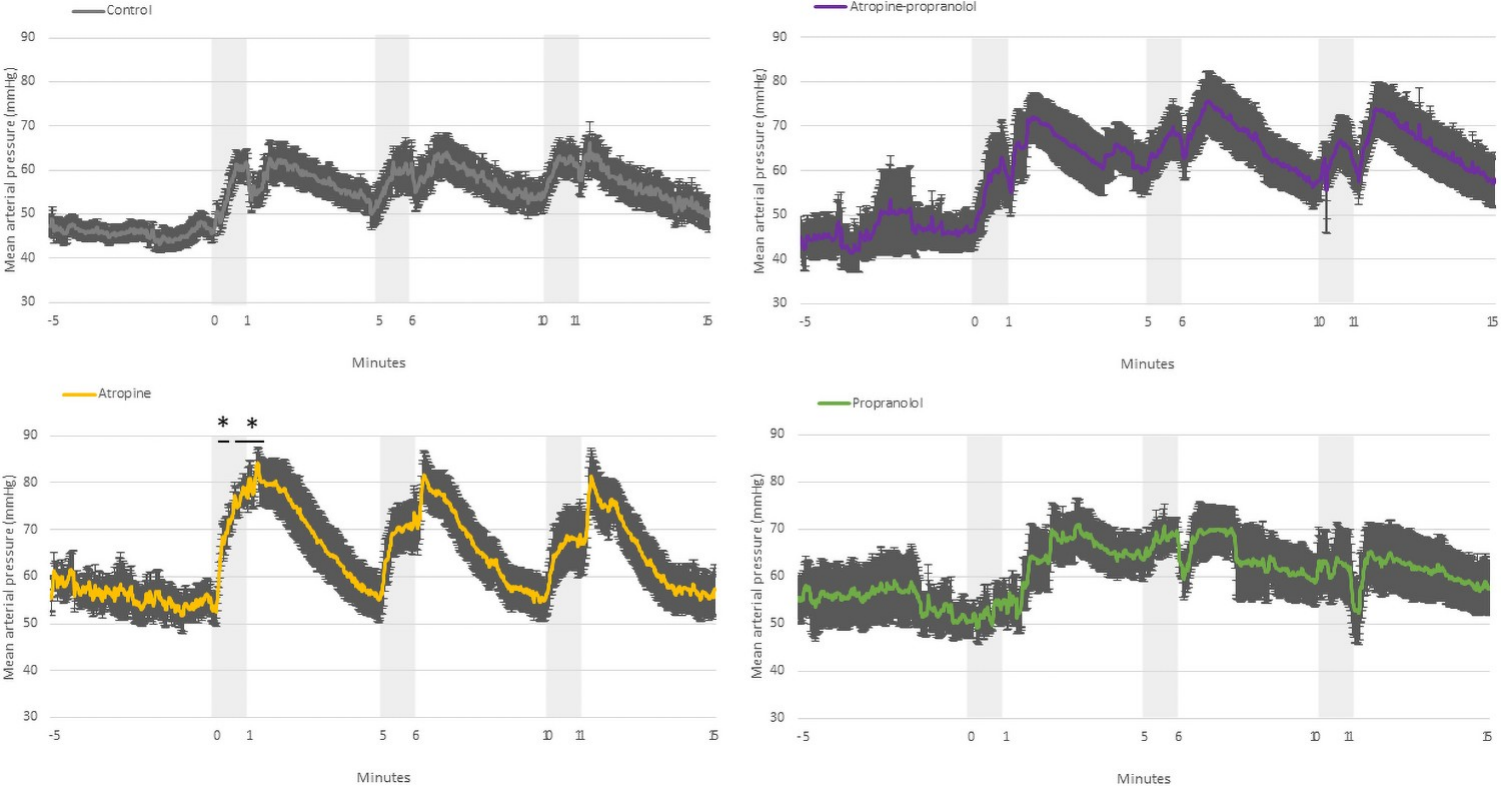
Change in fetal heart rate. Change in fetal heart rate in control, atropine, propranolol and atropine-propranolol protocols in fetuses exposed to three applications of 1-minute umbilical cord occlusion (grey zone) every 5 minutes. Data are expressed as mean ± standard error. *p < 0.05 vs. control. Resolution of each datapoint: 1 second.

**Fig 5:**
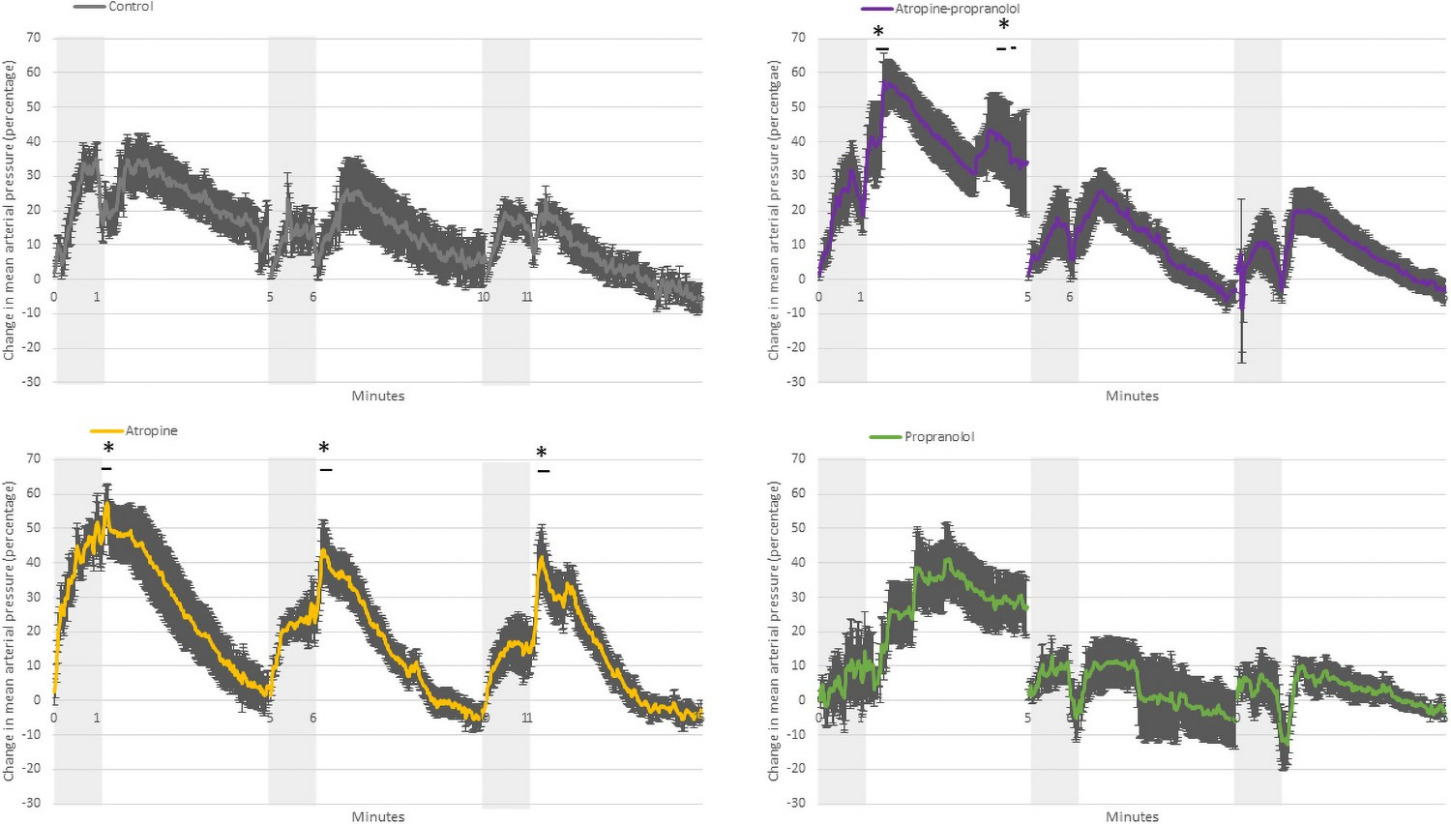
Change in fetal mean arterial pressure. Change in fetal mean arterial pressure in control, atropine, propranolol and atropine-propranolol protocols in fetuses exposed to three applications of 1-minute umbilical cord occlusion (grey zone) every 5 minutes. Data are expressed as mean ± standard error. *p < 0.05 vs. control. Resolution of each datapoint: 1 second.

*Control protocol*. UCO was associated with rapid bradycardia (p<0.0001) and hypertension (p<0.0001) (Fig 1).

*Atropine protocol*. FHR slowly decreased during UCO (p<0.01), while MAP was higher than that at baseline (p<0.0001) (Fig 1). FHR was markedly higher compared with controls during all three occlusions (Fig 2). The change in FHR was significantly lower with the atropine protocol than with the control protocol, except during the latter stage of the first occlusion (Fig 3). The absolute rate of increase in MAP was higher in the atropine group compared with controls during the first occlusion (Fig 4). However, the change in MAP was similar between the atropine and control protocols (Fig 5).

*Propranolol protocol*. Bradycardia occurred compared to the baseline (p<0.0001), while MAP was similar to baseline (Fig 1). Occlusions were associated with a greater reduction in FHR only during the second and third occlusions (Fig 2). The change in FHR was similar between the propranolol and control protocols (Fig 3). The MAP and the change in MAP were similar between the propranolol and control protocols (Fig 5).

*Atropine-propranolol protocol*. FHR decreased progressively during UCO (p<0.0001), whereas MAP increased (p<0.001) (Fig 1). FHR was markedly higher compared with controls during all three occlusions, except during the end of the first and third occlusions (Fig 2). The change in FHR was significantly smaller with the atropine-propranolol protocol than with the control protocol during the early stage of the three occlusions (Fig 3). The MAP and the change in MAP were similar between the atropine-propranolol and control protocols (Figs 4 and 5).

#### Interocclusion periods (Figs 1 to 5)

*Control protocol*. FHR remained lower than baseline for up to 120 s (p<0.001) and gradually returned to near-baseline levels; MAP remained elevated during the first part of recovery (p<0.001), and gradually returned to baseline levels (Fig 1).

*Atropine protocol*. FHR progressively returned to baseline after an overshoot up to 120 s (p<0.001). MAP remained elevated up to 120 s (p<0.001), and progressively returned to baseline (Fig 1). FHR and the change in FHR were markedly higher than controls (Figs 2 and 3). MAP was only higher in atropine protocol than with the control protocol during the beginning of the first interocclusion period (Fig 4). The change in MAP was greater with the atropine protocol than with the control protocol during several seconds at the early stage of the interocclusion periods (Fig 5).

*Propranolol protocol*. FHR progressively returned to baseline, while MAP was always similar to baseline (Fig 1). FHR was markedly higher compared with controls during all interocclusion periods (Fig 2). Change in FHR was transiently lower than control protocol during the first and second interocclusion periods (Fig 3). The MAP and the change in MAP were similar between the propranolol and control protocols (Figs 4 and 5).

*Atropine-propranolol protocol*. FHR progressively returned to baseline. MAP remained elevated and gradually returned to baseline (Fig 1). FHR was markedly higher compared with controls during all interocclusion periods (Fig 2). Change in FHR was transiently higher than control protocol during the beginning of the first interocclusion period (Fig 3). The MAP was similar between the atropine-propranolol and control protocols (Fig 4). The change in MAP was only higher during several seconds of the first interocclusion period (Fig 5).

### Fetal biological parameters

Blood gases (pH, PaCO_2_, PaO_2_) and plasma lactate concentrations was similar between the four protocols at the end of three applications of UCO (Table 2).

**Table 2:** Comparison of the concentrations of fetal arterial blood gases and lactate between protocols at the end of the three cord occlusions.

Blood gases/Protocols	Control	Atropine	Propranolol	Atropine-propranolol	p-value
pH	7.28 (7.15–7.30)	7.26 (7.15–7.29)	7.21 (7.20–7.23)	7.21 (7.20–7.26)	0.7
PaCO_2_ (mmHg)	50.3 (46.9–55.6)	45.5 (43.9–49.4)	57.7 (54.7–58.1)	53.2 (52.1–59.5)	0.11
PaO_2_ (mmHg)	15 (13–18)	16 (15–17)	17 (14–18)	15 (15–17)	0.79
Lactates (mmol/L)	6.19 (4.73–9.18)	5.66 (5.12–5.88)	6.11 (5.08–7.63)	6.53 (6.02–6.63)	0.73

## Discussion

Deceleration of FHR is common during labor and reflects fetal compensation in response to hypoxia during uterine contractions [2]. The present study examined our understanding of fetal cardiovascular adaptation and the role of the ANS during 1-minute complete UCO and during the recovery period.

Our results describe the respective role of the parasympathetic and sympathetic systems during and after brief but complete UCO. Cord compression induces a rapid decrease in the FHR and a rapid increase in MAP. The decrease in FHR is caused by an increase in parasympathetic activity, because we have shown that atropine and atropine-propranolol abolish the FHR response to UCO. The change in FHR was not modified by propranolol injection (no modification of change in FHR), showing no effect of sympathetic tone in controlling the FHR during occlusion. The increase in MAP during UCO was similar in the four protocols. After releasing UCO, the FHR was still lower than that at baseline due to a sustained parasympathetic tone. Suppression of the parasympathetic output to the cardiovascular system unmasks an increase in the FHR above baseline values. The lower FHR with the propranolol protocol further supports an increase in myocardial β-adrenoceptor stimulation after cord release. Four minutes after UCO release, the FHR returned to baseline irrespective of the drugs that were infused, thereby showing recovery of ANS control.

Taken together, these results indicate that the brief and complete UCO-induced hemodynamic response results mainly from acute activation of the parasympathetic and sympathetic output. Our data confirm that during complete UCO, the key role of parasympathetic tone is in controlling the FHR, and during the interocclusion period, the key role of sympathetic and parasympathetic tone is in controlling FHR. However, we found no effect due to the sympathetic tone in controlling the FHR during UCO. After releasing UCO, parasympathetic output was elevated and myocardial β-adrenoceptors were stimulated in controlling the FHR. Moreover, we found no effect of sympathetic and parasympathetic tone in controlling MAP during 1-minute UCO and during the interocclusion period. However, parasympathetic tone is involved in controlling MAP during the early stage of the interocclusion periods.

Lear et al. have discussed the physiology of heart rate decelerations and the involvement of several reflexes [3]. Activation of the chemoreflex can lead to FHR deceleration and increase in MAP via peripheral (carotid and aortic body) and central chemoreceptors, which increase both sympathetic and parasympathetic activities [15]. The chemoreflex is triggered by the decrease of PaO_2_ or pH, or an increase in PaCO_2_ [15,16]. Itskovitz et al. report significant reductions in PO2 during 25, 50, 75 and 100% UCOs of 40 seconds in duration [17]. Several experimental studies of brief repeated asphyxia in fetal sheep have shown decrease in PaO_2_ after a few minute of asphyxia [5,6,18,19]. Smolich et al., in two animal studies, found a marked fall in oxygen levels during occlusion, occurring at 15 seconds [20,21]. Previous studies have highlighted that chemoreflex activation is delayed by several seconds after the carotid body have sensed a change in PaO_2_, [16,22]. This further delays the possible activation of the chemoreflex when chemoreceptors have sensed a change in blood gases. As described by Lear et al., the first 3–4 seconds of the brief-UCO are consistent with a baroreflex. However, they have found that the remainder of the decelerations are consistent with the peripheral chemoreflex [23]. In Künzel’s study, fetal saturation decreased during complete cord occlusion [24]. Lear et al. has shown that the cerebral oxygenation, measured as the difference between oxyhemoglobin and deoxyhemoglobin (called delta-hemoglobin), fell in parallel with FHR until the FHR nadir [25]. Oxygenation may decrease despite no change in preductal PaO_2_ through a drop in cardiac output. However, as demonstrated by previous studies, cortical blood flow (based on laser Doppler) and carotid blood flow remains stable for several minutes during even prolonged UCO [26,27]. Moreover, previous studies reported that the main stimulus of peripheral chemoreceptors at the carotid and aortic body is the fall in PaO_2_ rather than O_2_ content (which is determined by the hemoglobin concentration and its saturation) [16,22]. Hunter et al. showed, in preterm neonates <37 weeks gestation population, no significant correlation between cerebral oxygenation and arterial oxygenation (PaO_2_) [28]. No similar studies have been conducted in fetuses. However, Hunter et al. found a rapidly fall in cortical tissue PaO_2_ during UCO [29]. In our study, PaO_2_ decrease at the end of the three occlusions. However, we did not perform blood gases during the 1-minute occlusion in each protocol. We did not perform cortical blood flow and measure of cortical tissue PaO_2_. The next stage will be to measure cortical blood flow and cortical tissue PaO_2_, and to explore the role of the chemoreflex during complete and brief-UCO.

During 1 minute of UCO, we saw no evidence of a change in myocardial β-adrenoceptor activity on the FHR. Lear et al. also found that the FHR was significantly lower in the propranolol group during all three UCOs and inter-occlusion periods [30]. Moreover, Galinsky et al showed a difference in deceleration between the control and propranolol groups [5]. However, the experimental procedure was different, as the umbilical cord was occluded for 2 min. In the Galinsky study, the difference in FHR between the two groups occur mainly after the first min of UCO or after successive UCO. However, in both studies, FHR baselines were different between the control and propranolol protocols. In our study, FHR was also expressed as % of change from baseline to compare the FHR changes between groups. Our data provide evidence that FHR change during the 1-min UCO is not influenced by β1-adrenoceptors activity. Lear et al. highlighted that sympathetic activation has limited effects on FHR during repeated episodes of brief UCO, indicating that the regulation of FHR may be due to other stimuli, such as adrenal catecholamines [10,21]. We did not measure blood catecholamines. There is evidence that adrenal catecholamine plasma concentrations increase during UCO [6,21]. However, the role of the adrenal catecholamines on the FHR is presently unclear, at least during the first min of the UCO. Chemoreflex activation is usually associated with an increase in sympathetic output to increase the MAP [6,15]. Galinsky et al. have concluded, in a model of sympathectomy using 6-hydroxydopamine (6-OHDA) with 2 minutes of UCO, that the sympathetic nervous system supports arterial blood pressure during prolonged exposure to brief repeated UCO with metabolic acidosis. They found that in the 6-OHDA group, only the first UCO was associated with an initial fall in MAP that was recorded over the remainder of the occlusion period. Thereafter, sympathectomy was associated with an initial rapid increase in MAP during occlusion, which was followed by a more rapid fall in MAP during the first 60 s of occlusion compared with controls [6]. The 6-OHDA impaired the rate of fall of femoral vascular conductance, i.e., the rate of femoral vasoconstriction. We did not study the role of the sympathetic nervous system on the peripheral vasoconstriction during UCO. In the Galinsky and the Lear studies, during 2 minutes of UCO, β-adrenoceptor blockade did not modify the MAP response during the first minute of UCO, but only during the second minute [5,30]. The increase in arterial pressure during UCO may be mediated by peripheral vasoconstriction, suggesting an increase in sympathetic tone or in adrenal catecholamines release, as α-adrenergic peripheral agonist. However, we did not evaluate α-adrenergic blockade in our study. Moreover, the combination of the present and previous studies, catecholamines have a greater role during more severe episodes of repeated hypoxia, but less role during moderate hypoxia as in this study. The inotropic role of myocardial β-adrenergic activity associated with α-adrenergic peripheral activities could explain the rise in MAP observed during 1-minute of UCO. Moreover, during UCO with the atropine protocol, Galinsky et al. observed a rise in MAP [5]. We similarly observed that MAP increased with both atropine and control protocols. They concluded that the rise of MAP in the atropine group reflects maintenance of the FHR and combined ventricular output. Smolich et al. have demonstrated, in the initial 30 seconds of UCO, that the left ventricular output did not decrease, contrary to the right ventricular output which fall by 20%, due to a reduced stroke volume associated with increased arterial blood pressures. Moreover, they have found that preload is not reduced, further strengthening the argument that changes in preload do not mediate intrapartum decelerations [21]. Immediately after UCO release, we found a higher rise in MAP with the atropine protocol than with the control protocol during the early stage of interocclusion period, which could be explained by the rise in FHR and combined ventricular output.

After releasing cord compression, both sympathetic activity and parasympathetic activity increased. Sixty seconds after cord release, MAP was still higher than that at baseline due to sustained stimulation of myocardial β-adrenoceptors, which propranolol prevented. Moreover, an increase in the parasympathetic tone was still present at that time because arterial pressure was higher after atropine injection than it was in control fetal lambs. At 4 minutes after occlusion, no difference in blood pressure was found among the protocols when observing for change in FHR, suggesting that ANS activity returned to baseline. Sympathetic activation may indicate chemoreflex activation during this period, as suggested by previous studies [5,6]. An increase in sympathetic activity may explain the sustained increase in blood pressure despite cord release and restoration of the low-resistance placental circulation [5,6,31]. Activation of α- and β-adrenoceptors may increase peripheral vasoconstriction and ventricular output [5,6].

Our results provide additional insight regarding the role of the ANS during labor and delivery. Variable decelerations in the FHR are commonly seen on the cardiotocogram, which is used to monitor fetal well-being [1,2]. Labor is characterized by intermittent and brief episodes of hypoxia (no more than 1 to 2 minutes) during uterine contractions [1,2]. These decelerations may result, at least in part, from umbilical cord compression, such as during cord prolapse. However, cord compression is unlikely to be the main cause of FHR decelerations in the majority of labors [7–9]. Indeed, fetal head compression has been reported, by Parer et al., to be responsible for early FHR decelerations, due to a transient change in cerebral blood flow resulting in vagal discharge [32]. They theorize that cord occlusion is likely the cause of many variable FHR decelerations in the first stage of active labor, and that head compression is responsible for decelerations in the second stage of labor [31]. However, Lear et al concluded that fetal head compression was unlikely to be a major contributor to intrapartum decelerations during the majority of labors [3,33]. However, as described by Parer and Lear, vagal reflex is involved in FHR deceleration whatever the etiology (umbilical cord compression or head compression or both) [32,33]. Understanding the nature of the reflexes involved in deceleration may help us understand what causes changes in fetal homeostasis and the response of the fetus. Sympathetic activity is essential for maintaining peripheral vasoconstriction and arterial pressure during repeated cord compression [5,10]. Parasympathetic activity is involved in the decrease in FHR, as observed in daily clinical practice. Understanding the physiological control of adaptation is of fundamental importance to recognizing when fetal autonomic adaptation is no longer efficient for the fetus.

Our study had several limits. First, despite many similarities existing between sheep and human gestation, the reproducibility of our observations and the extrapolation to the human fetus must be considered with caution. Second, although our model did not use or accurately represent a specific insult during labor, we evaluated FHR deceleration in UCO using methods described by others [5,6,10,17]. Even if UCO is one of the causes of fetal bradycardia, the conclusion may be interpreted only in the case of complete and brief UCO during labor, particularly during cord prolapse. Other mechanisms have been described, such as placenta or head compression, but the conclusions cannot be extrapolated to this present study [7,8]. Moreover, we did not evaluate the effect of uterine contractions on the onset of FHR decelerations, as observed in human fetuses, or the role of uterine contractions on sympathetic or parasympathetic stimulation during labor. Indeed, uterine contractions could induce a sympathetic response in fetuses to prevent heart rate decelerations, as manifested by an acceleration during the contraction. The sheep fetus has no forewarning of the occlusion, no premonitory increase in intrauterine pressure or option for anticipatory sympathetic output. It would be important to illustrate these responses in the experimental animals as well. Moreover, we did not study the vasomotor component, which would provide greater insight of reflexes. In addition, before occlusion, lactate levels were between 1 and 2 mmol/l. However, other parameters were normal, and the literature reported the same lactate levels [34]. In addition, we did not study the vasomotor component. We did not perform protocol with α-blockade. Moreover, we did not attempt to distinguish between the reflexes; the aim was to examine the role of the parasympathetic and sympathetic activity in changes in FHR and in arterial pressure during UCO. Finally, we did not perform blood gases during UCO.

## Conclusion

This study investigated the role of parasympathetic activity and sympathetic activity in FHR and arterial pressure during and after UCO. One minute of UCO-induced FHR deceleration was caused by an elevation in parasympathetic activity. Furthermore, we showed that myocardial β-adrenoceptor activity, which is involved in the increase in MAP. These results indicate that it is possible that the baroreflex and chemoreflex are not the only reflexes involved during the early phase of UCO-induced FHR deceleration. Several methods use FHR variability, which reflects ANS control of the sinus node, to assess fetal well-being [35,36]. Further studies are required to gain a better understanding of how the fetal autonomic system adapts over time to decreases in PaO_2_ and to hypoxia after repeated UCO.
